# DeepNano: Deep recurrent neural networks for base calling in MinION nanopore reads

**DOI:** 10.1371/journal.pone.0178751

**Published:** 2017-06-05

**Authors:** Vladimír Boža, Broňa Brejová, Tomáš Vinař

**Affiliations:** Faculty of Mathematics, Physics and Informatics, Comenius University in Bratislava, Slovakia; University of Texas Health Science Center at Houston, UNITED STATES

## Abstract

The MinION device by Oxford Nanopore produces very long reads (reads over 100 kBp were reported); however it suffers from high sequencing error rate. We present an open-source DNA base caller based on deep recurrent neural networks and show that the accuracy of base calling is much dependent on the underlying software and can be improved by considering modern machine learning methods. By employing carefully crafted recurrent neural networks, our tool significantly improves base calling accuracy on data from R7.3 version of the platform compared to the default base caller supplied by the manufacturer. On R9 version, we achieve results comparable to Nanonet base caller provided by Oxford Nanopore. Availability of an open source tool with high base calling accuracy will be useful for development of new applications of the MinION device, including infectious disease detection and custom target enrichment during sequencing.

## Introduction

In this paper, we introduce DeepNano, an open-source base caller for the MinION nanopore sequencing platform. The MinION device by Oxford Nanopore [1], weighing only 90 grams, is currently the smallest high-throughput DNA sequencer. Thanks to its low capital costs, small size and the possibility of analyzing the data in real time as they are produced, MinION is very promising for clinical applications, such as monitoring infectious disease outbreaks [2–4], and characterizing structural variants in cancer [5]. Here, we consider two versions of MinION technology, R7.3 and R9. The latter was introduced in May 2016.

Although MinION is able to produce long reads, data produced on the R7.3 version of the platform exhibit a rather high sequencing error rate. In this paper, we show that this error rate can be significantly reduced by improving the base caller. Moreover, an open-source base caller with good performance is essential for developing novel applications of MinION devices that require modifications of standard base calling utilities. Recent examples of such applications include runtime read selection protocols [6] and determination of complex methylation patterns in certain genomes [7].

**Fig 1:**
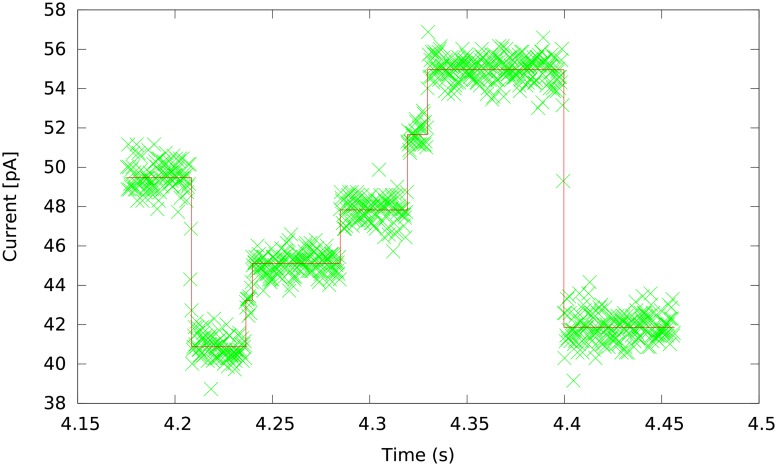
Raw signal from MinION and its segmentation to events. The plot was generated from the *E. coli* data (http://www.ebi.ac.uk/ena/data/view/ERR1147230).

In the MinION device, single-stranded DNA fragments move through nanopores, which causes changes in the electric current. The electric current is measured at each pore thousands times per second, resulting in a measurement plot as shown in Fig 1. The electric current depends mostly on the context of several DNA bases passing through the pore at the time of measurement. As the DNA moves through the pore, the context shifts and the electric current changes. Based on these changes, the sequence of measurements is split into *events*, each event ideally representing the shift of the context by one base. Each event is summarized by the mean and variance of the current and by event duration. This sequence of events is then translated into a DNA sequence by a base caller.

A MinION device typically yields reads several thousand bases long; reads as long as 100,000 bp have been reported. To reduce the error rate, the device attempts to read both strands of the same DNA fragment. The resulting template and complement reads can be combined to a single two-directional (2D) read during base calling. As shown in Table 1, this can reduce the error rate of the default base caller from roughly 30% for 1D reads to 13-15% for 2D reads on the R7.3 version.

**Table 1:** Accuracy of base callers on two R7.3 testing data sets. The results of base calling were aligned to the reference using BWA-MEM [28]. The accuracy was computed as the number of matches in the alignment divided by the length of the alignment.

	*E. coli*	*K. pneumoniae*
**Template reads**		
Metrichor	71.3%	68.1%
Nanocall	68.3%	67.5%
DeepNano	77.9%	76.3%
**Complement reads**		
Metrichor	71.4%	69.5%
Nanocall	68.5%	68.4%
DeepNano	76.4%	75.7%
**2D reads**		
Metrichor	86.8%	84.8%
DeepNano	88.5%	86.7%

Oxford Nanopore’s default basecaller was provided on the Metrichor cloud computing platform (called “Metrichor” here for short). It is a proprietary software, and the exact details of its algorithms are not known. Metrichor for R7.3 platform assumed that each event depends on a context of *k* = 6 consecutive bases and that the context typically shifts by one base in each step. As a result, every base is read as a part of *k* consecutive events. This process can be represented by a hidden Markov model (HMM). Each state in the model represents one *k*-tuple and the transitions between states correspond to *k*-tuples overlapping by *k* − 1 bases (e.g. AACTGT will be connected to ACTGTA, ACTGTC, ACTGTG, and ACTGTT), similarly as in de Bruijn graphs. Emission probabilities reflect the current expected for a particular *k*-tuple, with an appropriate variance added. Finally, additional transitions represent missed events, falsely split events, and other likely errors (in fact, insertion and deletion errors are quite common in the MinION sequencing reads, perhaps due to errors in event segmentation). After parameter training, base calling can be performed by running the Viterbi algorithm, which will result in the sequence of states with the highest likelihood. It is not known, what is the exact nature of the model used in Metrichor, but the emission probabilities required for this type of model are provided by Oxford Nanopore in the files storing the reads. The same approach has also been implemented in an open source base caller Nanocall [8], which was released simultaneously with an early version of DeepNano (arXiv:1603.09195).

There are several disadvantages to using HMMs for base calling of MinION data. HMMs are very good at representing short-range dependencies (such as moving from one *k*-mer to the next), yet it has been hypothesized that long-range dependencies may also play a role in MinION base calling, and such dependencies are very hard to capture in HMMs. Also, in HMMs a prior model for the DNA sequence itself is a part of the model. This may be difficult to provide for an unknown DNA sequence and using incorrect prior model may cause significant biases.

Our base caller uses recurrent neural networks, which have stellar results for speech recognition [9], machine translation [10], language modeling [11], and other sequence processing tasks. The current version of Metrichor is also based on recurrent neural networks. Neural networks were previously used for base calling Sanger sequencing reads [12, 13], though the nature of MinION data is rather different.

Several tools for processing nanopore sequencing data were already published, including read mappers [14, 15], and error correction tools using short Illumina reads [16]. Most closely related to our work are Nanopolish [17] and PoreSeq [18]. Both tools create a consensus sequence by combining information from multiple overlapping reads, considering not only the final base calls from Metrichor, but also the sequence of events. They analyze the events by hidden Markov models with emission probabilities provided by Metrichor. In contrast, our base caller does not require read overlaps, it processes reads individually and provides more precise base calls for downstream analysis. The crucial difference, however, is our use of a more powerful framework of recurrent neural networks. Thanks to a large hidden state space, our network can potentially capture long-distance dependencies in the data, whereas HMMs use fixed *k*-mers.

## Methods

In this section, we describe the design of our base caller, which is based on deep recurrent neural networks. A thorough coverage of modern methods in deep learning can be found in [19]. A recurrent neural network [20, 21] is a type of artificial neural network used for sequence labeling. Given a sequence of input vectors $\left\{{\vec{x}}_{1},{\vec{x}}_{2},\ldots ,{\vec{x}}_{t}\right\}$, its prediction is a sequence of output vectors $\left\{{\vec{y}}_{1},{\vec{y}}_{2},\ldots ,{\vec{y}}_{t}\right\}$. In our case, each input vector ${\vec{x}}_{i}$ consists of the mean, standard deviation and length of each event, and the output vector ${\vec{y}}_{i}$ gives a probability distribution of called bases.

**Simple recurrent neural networks.** First, we describe a simple recurrent neural network with one hidden layer. During processing of each input vector ${\vec{x}}_{i}$, a recurrent neural network calculates two vectors: its hidden state ${\vec{h}}_{i}$ and the output vector ${\vec{y}}_{i}$. Both depend on the current input vector and the previous hidden state: ${\vec{h}}_{i}=f\left({\vec{h}}_{i-1},{\vec{x}}_{i}\right)$, ${\vec{y}}_{i}=g\left({\vec{h}}_{i}\right)$. We will describe our choice of functions *f* and *g* later. The initial state ${\vec{h}}_{0}$ is one of the parameters of the model.

Prediction accuracy can be usually improved by using neural networks with several hidden layers, where each layer uses hidden states from the previous layer. We use networks with three or four layers. Calculation for three layers proceeds as follows:

$$\begin{array}{ccc} {\vec{h}}_{i}^{\left(1\right)} & = & f_{1}\left({\vec{h}}_{i-1}^{\left(1\right)},{\vec{x}}_{i}\right)\\ {\vec{h}}_{i}^{\left(2\right)} & = & f_{2}\left({\vec{h}}_{i-1}^{\left(2\right)},{\vec{h}}_{i}^{\left(1\right)}\right)\\ {\vec{h}}_{i}^{\left(3\right)} & = & f_{3}\left({\vec{h}}_{i-1}^{\left(3\right)},{\vec{h}}_{i}^{\left(2\right)}\right)\\ {\vec{y}}_{i} & = & g({\vec{h}}_{i}^{\left(3\right)}) \end{array}$$

Note that in different layers, we use different functions *f*_1_, *f*_2_, and *f*_3_, where each function has its own set of parameters.

**Fig 2:**
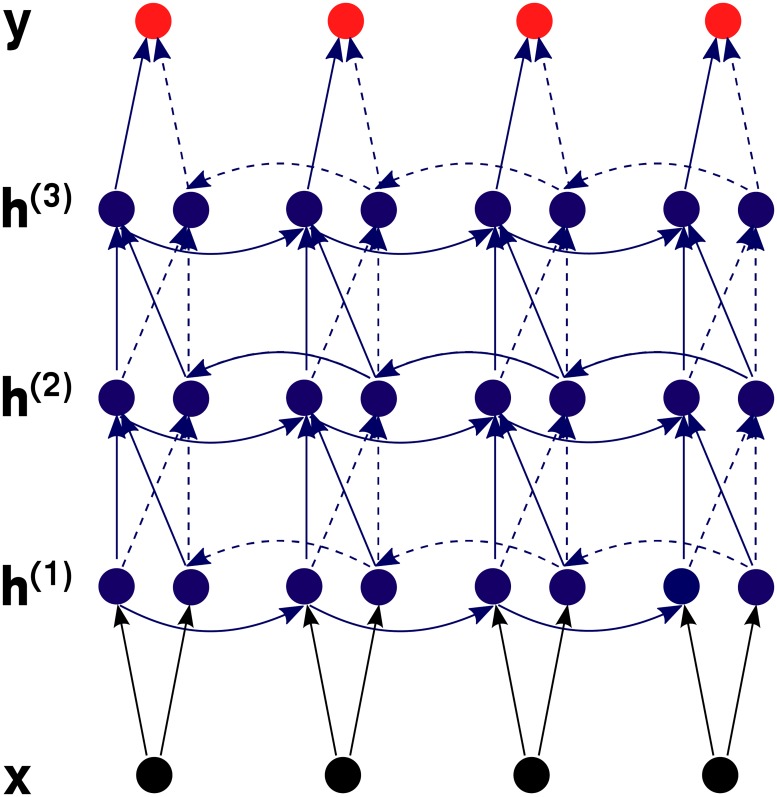
Schematics of a bidirectional recurrent neural network.

**Bidirectional recurrent neural networks.** In our case, the prediction for input vector ${\vec{x}}_{i}$ can be influenced by data seen before ${\vec{x}}_{i}$ but also by data seen after it. To incorporate this data into prediction, we use a bidirectional neural network [22], which scans data in both directions and concatenates hidden outputs before proceeding to the next layer (see Fig 2). Thus, for a two-layer network, the calculation would proceed as follows (|| denotes concatenation of vectors):

$$\begin{array}{ccc} {\vec{h}}_{i}^{\left(1+\right)} & = & f_{1+}\left({\vec{h}}_{i-1}^{\left(1+\right)},{\vec{x}}_{i}\right)\\ {\vec{h}}_{i}^{\left(1-\right)} & = & f_{1-}\left({\vec{h}}_{i+1}^{\left(1-\right)},{\vec{x}}_{i}\right)\\ {\vec{h}}_{i}^{\left(1\right)} & = & {\vec{h}}_{i}^{\left(1+\right)}\left|\right|{\vec{h}}_{i}^{\left(1-\right)}\\ {\vec{h}}_{i}^{\left(2+\right)} & = & f_{2+}\left({\vec{h}}_{i-1}^{\left(2+\right)},{\vec{h}}_{i}^{\left(1\right)}\right)\\ {\vec{h}}_{i}^{\left(2-\right)} & = & f_{2-}\left({\vec{h}}_{i+1}^{\left(2-\right)},{\vec{h}}_{i}^{\left(1\right)}\right)\\ {\vec{h}}_{i}^{\left(2\right)} & = & {\vec{h}}_{i}^{\left(2+\right)}\left|\right|{\vec{h}}_{i}^{\left(2-\right)}\\ {\vec{y}}_{i} & = & g({\vec{h}}_{i}^{\left(2\right)}) \end{array}$$

**Gated recurrent units.** The typical choice of function *f* in a recurrent neural network is a linear transformation of inputs followed by hyperbolic tangent nonlinearity:

$$f\left({\vec{h}}_{i-1},\vec{x_{i}}\right)=\tanh\left(W\vec{x_{i}}+U{\vec{h}}_{i-1}+\vec{b}\right),$$

where the matrices *W*, *U* and the bias vector $\vec{b}$ are the parameters of the model.

This choice unfortunately leads to the vanishing gradient problem [23]. During parameter training, the gradient of the error function in layers further from the output is much smaller that in layers closer to the output. In other words, gradient diminishes during backpropagation through the network, complicating parameter training.

One of the solutions is to use a network with gated recurrent units [24]. Given input $\vec{x_{i}}$ and previous hidden state ${\vec{h}}_{i-1}$, a gated recurrent unit first calculates values for update and reset gates:

$${\vec{u}}_{i}=\sigma \left(W_{u}\vec{x_{i}}+U_{u}{\vec{h}}_{i-1}+{\vec{b}}_{u}\right),$$

$${\vec{r}}_{i}=\sigma \left(W_{r}\vec{x_{i}}+U_{r}{\vec{h}}_{i-1}+{\vec{b}}_{r}\right),$$

where *σ* is the sigmoid function: *σ*(*z*) = 1/(1 + *e*^−*z*^). Then the unit computes a potential new value

$${\vec{n}}_{i}=\tanh\left(W\vec{x_{i}}+\vec{r_{i}}\circ U{\vec{h}}_{i-1}\right).$$

Here, ∘ is the element-wise vector product. If some component of the reset gate vector is close to 0, it decreases the impact of the previous state.

Finally, the overall output is a linear combination of ${\vec{n}}_{i}$ and ${\vec{h}}_{i-1}$, weighted by the update gate vector $\vec{u_{i}}$:

$${\vec{h}}_{i}=\vec{u_{i}}\circ {\vec{h}}_{i-1}+\left(1-{\vec{u}}_{i}\right)\circ {\vec{n}}_{i}.$$

Note that both gates give values from interval (0, 1) and allow for a better flow of the gradient through the network, making training easier.

Matrices *W*_*u*_, *U*_*U*_, *W*_*r*_, *U*_*r*_, *W*, *U*, and vectors ${\vec{b}}_{u}$, ${\vec{b}}_{r}$ are parameters of our model estimated by training. Note that we use separate parameters for each layer and direction of the network. In a layer with inputs ${\vec{h}}_{i-1}$ of length *n*_1_ and ${\vec{x}}_{i}$ of length *n*_2_, and output ${\vec{h}}_{i}$ of length *m*, matrices *W*_*u*_, *W*_*r*_, and *W* have size *m* × *n*_2_, matrices *U*_*u*_, *U*_*r*_, and *U* have size *m* × *n*_1_, and vectors ${\vec{b}}_{u}$, ${\vec{b}}_{r}$ have length *m*. For example, in a network with 100 hidden units per layer, the first layer has *n*_1_ = *m* = 100 and *n*_2_ = 3, the middle layers have *n*_1_ = *n*_2_ = *m* = 100 and the final layer has *n*_1_ = *n*_2_ = 100 and *m* = 10.

**Output layer.** Typically, one input event leads to one called base. But sometimes we get multiple events for one base, so there is no output for some events. Conversely, some events are lost, and we need to call multiple bases for one event. We limit the latter case to two bases per event. For each event, we output two probability distributions over the alphabet Σ = {*A*, *C*, *G*, *T*, −}, where the dash means no base. We will denote the two bases predicted for input event ${\vec{x}}_{i}$ as $b_{i}^{\left(1\right)}$ and $b_{i}^{\left(2\right)}$. Probability of each base *q* ∈ Σ is calculated from the hidden states in the last layer using the softmax function:

$$P\left[b_{i}^{\left(k\right)}=q\right]=\frac{\exp({\vec{\theta }}_{q}^{\left(k\right)}{\vec{h}}_{i}^{\left(3\right)})}{\sum_{p\in \Sigma }\exp\left({\vec{\theta }}_{p}^{\left(k\right)}{\vec{h}}_{i}^{\left(3\right)}\right)}.$$

Vectors ${\vec{\theta }}_{q}^{\left(k\right)}$ for *k* = 1, 2 and *q* ∈ Σ are also parameters of the model.

Final base calling is done by taking the most probable base for each $b_{i}^{\left(k\right)}$ (or no base if dash is the most probable character from Σ). During training, if there is one base per event, we always set $b_{i}^{\left(1\right)}$ to dash.

During our experiments, we found that outputing two independent distributions works better than outputing one distribution with 21 symbols (nothing, 4 options for one base, 16 options for two bases).

**Training.** Let us first consider the scenario in which we know the correct DNA bases for each input event. The goal of the training is then to find parameters of the network that maximize the log likelihood of the correct outputs. In particular, if $o_{1}^{\left(1\right)},o_{1}^{\left(2\right)},o_{2}^{\left(1\right)},\ldots ,o_{n}^{\left(1\right)},o_{n}^{\left(2\right)}$ is the correct sequence of output bases, we try to maximize the sum

$$\sum_{i=1}^{n}\mathrm{lg}P\left[b_{i}^{\left(1\right)}=o_{1}^{\left(1\right)}\right]+\mathrm{lg}P\left[b_{i}^{\left(2\right)}=o_{2}^{\left(2\right)}\right]$$

As an optimization algorithm, we use stochastic gradient descent (SGD) combined with Nesterov momentum [25] to increase the convergence rate. For 2D base calling, we first use SGD with Nesterov momentum, and after several iterations we switch to L-BFGS [26]. Our experience suggests that SGD is better at avoiding bad local optima in the initial phases of training, while L-BFGS seems to be faster during the final fine-tuning.

Unfortunately, we do not know the correct output sequence; more specifically, we only know the region of the reference sequence where the read is aligned, but we do not know the exact pairs of output bases for individual events. We solve this problem in an EM-like fashion.

First, we create an approximate alignment between the events and the reference sequence using a simple heuristic. With R7.3 data, the expected signal means for *k*-mer contexts were readily provided by Oxford Nanopore. Using this data, one can simulate the expected signal from the reference sequence and then use a simple dynamic programming to find an alignment that minimizes the sum of differences between the expected and observed signal means, with simple penalties for splitting or skipping an event. With R9 data, we have simply used Metrichor base calls as the starting event alignment. The reason for this change is that it has become more difficult to obtain the parameters for simulating the signal, and at the same time, the Oxford Nanopore base callers have improved so that it is now possible to use their alignments as a starting point without a negative impact on the training process.

After each hundredth pass through the whole data set, we realign the events to the reference sequence by using the outputs from the RNN. We can interpret these outputs as posterior base probabilities. To each event, zero, one, or two reference sequence bases are aligned and the score of this alignment is the sum of logarithms of the corresponding output probabilities. Alignment maximizing this score can again be found by a simple dynamic programming.

**Data preprocessing.** The mean and standard deviation of measured signals change over time. The simplest way of accounting for this factor is to scale the measured values.

In our model, we can use scaling parameters calculated by Metrichor. In particular, we use scale and shift for mean, and scaling for standard deviation; we do not use drift for means as the use of this parameter has a negligible effect on the performance.

To make our approach independent of Metrichor, we have also implemented a simple method for computing scaling parameters. In particular, we set the scaling parameters so that the 25th and 75th percentile of the mean values fit predefined values and the median of the standard deviations fits a predefined value. Using either Metrichor scaling parameters or our simplified scaling yields a very similar performance in our experiments for R7.3 data. For R9 data, we have always used our percentile-based scaling.

**1D base calling.** The neural networks described above can be used for base calling template and complement strands in a straightforward way. Note that we need a separate model for each strand, since they have different properties. In both models, we use neural networks with three hidden layers and 100 hidden units.

**2D base calling.** In 2D base calling, we need to combine information from separate event sequences for the template and complement strands. A simple option is to apply neural networks for each strand separately, producing two sequences of output probability distributions. Then we can align these two sequences of distributions by dynamic programming and produce the DNA sequence with maximum likelihood.

However, this approach leads to unsatisfactory results in our models, with the same or slightly worse accuracy than the original Metrichor base caller. We believe that this phenomenon occurs because our models output independent probabilities for each base, while the Metrichor base caller allows dependencies between adjacent base calls.

Therefore, we have built a neural network which gets as an input corresponding events from the two strands and combines them to a single prediction. To do so, we need an alignment of the two event sequences, as some events can be falsely split or missing in one of the strands. We can use either the alignment obtained from the base call files produced by Metrichor or our own alignment, computed by a simple dynamic programming over output probabilities, which finds the path with the highest likelihood. We convert each pair of aligned events to a single input vector. Events present in only one strand are completed to a full input vector by special values. This input sequence is then used in a neural network with four hidden layers and 250 hidden units in each layer; we needed to use a bigger network than in 1D case since there is more information to process.

**Implementation details.** We have implemented our network using Theano library for Python [27], which includes symbolic differentiation and other useful features for training neural networks. We do not use any regularization, as with the size of our dataset we saw almost no overfitting.

## Results

**Data sets.** We have used existing data sets from *Escherichia coli* (http://www.ebi.ac.uk/ena/data/view/ERR1147230) and *Klebsiella pneumoniae* (http://www.ebi.ac.uk/ena/data/view/SAMEA3713789) by the SQK-MAP006 sequencing protocol with R7.3 flow cells. We have only used the reads that passed the original base calling process and had a full 2D base call. We have also omitted reads that did not map to the reference sequence (mapping was done separately for 2D base calls and separately for base calls from individual strands).

We have split the *E. coli* data set into training and testing portions; the training set contains the reads mapping to the first 2.5 Mbp of the genome. We have tested the predictors on reads which mapped to the rest of the *E. coli* genome and on reads from *K. pneumoniae*. Basic statistics of the two data sets are shown in Table 2.

**Table 2:** Sizes of experimental data sets. The sizes differ between strands because only base calls mapping to the reference were used. Note that the counts of 2D events are based on the size of the alignment.

	*E. coli* training	*E. coli* testing	*K. pneumoniae* testing
# of template reads	3,803	3,942	13,631
# of template events	26,403,434	26,860,314	70,827,021
# of complement reads	3,820	3,507	13,734
# of complement events	24,047,571	23,202,959	67,330,241
# of 2D reads	10,278	9,292	14,550
# of 2D events	84,070,837	75,998,235	93,571,823

**Accuracy comparison.** We have compared our base calling accuracy with the accuracy of the original Metrichor HMM base caller and with Nanocall [8] on two R7.3 testing data sets. The main experimental results are summarized in Table 1. We see that in the 1D case, our base caller is significantly better on both strands and in both data sets. In 2D base calling, our accuracy is still slightly higher than Metrichor.

On the *Klebsiella pneumoniae* data set, we have observed a difference in the GC content bias between different programs. This genome has GC content of 57.5%. DeepNano has underestimated the GC content on average by 1%, whereas the Metrichor base caller underestimated it by 2%.

**Fig 3:**
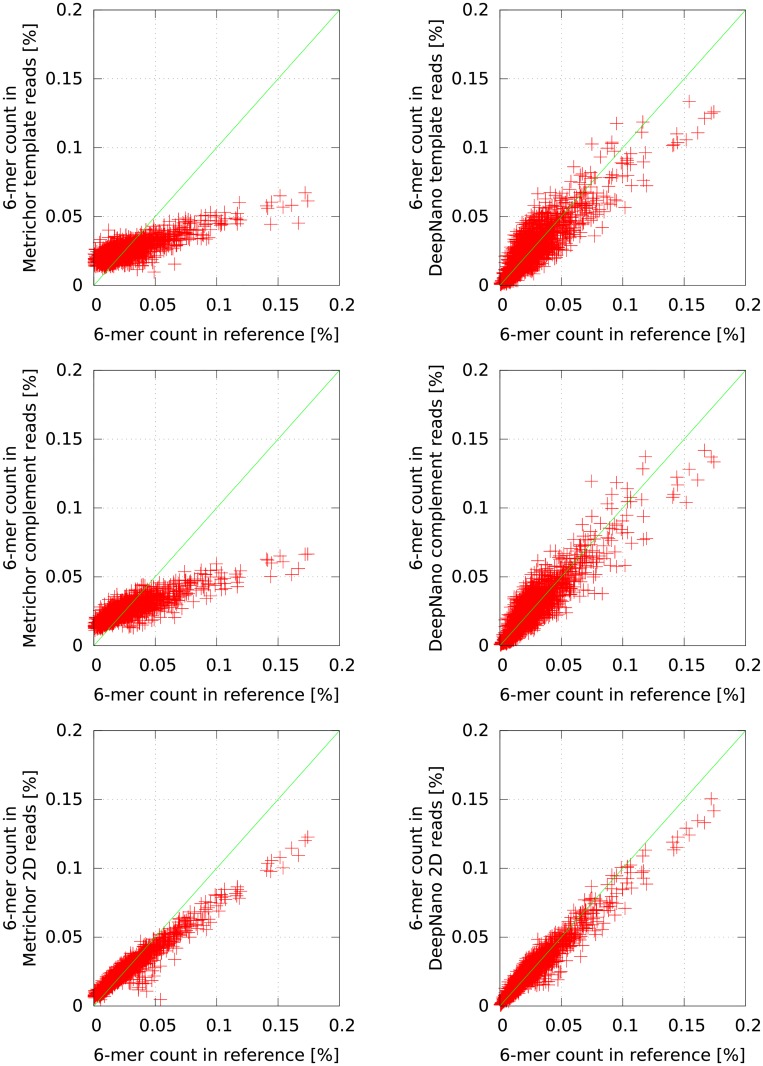
DeepNano reduces bias in 6-mer composition. Comparison of 6-mer content in *Klebsiella* reference genome and base-called reads by Metrichor (left) and DeepNano (right). From top to bottom: template, complement, 2D.

**Fig 4:**
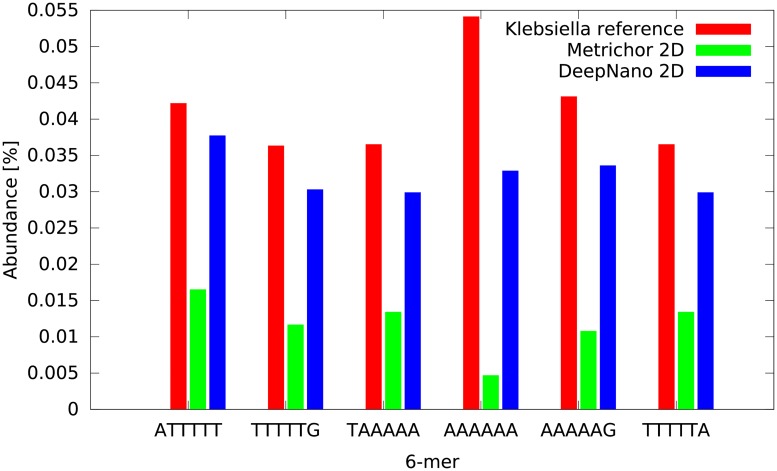
Abudances for repetitive 6-mers.

To explore sequence biases in more detail, we also compared the abundance of all 6-mers in the *Klebsiella* genome in the base-called reads. Fig 3 shows that base calls produced by DeepNano exhibits significantly smaller bias in 6-mer composition than Metrichor base calls. This trend is particularly pronounced for repetitive 6-mers (Fig 4); a similar bias was previously observed by [17].

With R9 version of the platform, Oxford Nanopore has introduced a variety of base calling options, including cloud-based Metrichor service, the local base calling option, experimental Nanonet code base, and binary-only Albacore platform, all of these options very similar in accuracy. We have used a benchmark *E. coli* data set from Loman lab (http://s3.climb.ac.uk/nanopore/R9_Ecoli_K12_MG1655_lambda_MinKNOW_0.51.1.62.tar) to evaluate performance of DeepNano and compare the accuracy to Nanonet, which is also based on RNNs. The training and testing sets were split in the same way as for R7.3 data sets. Table 3 shows that the accuracy of DeepNano is very similar to that of Nanonet, but DeepNano is faster than Nanonet. By decreasing the number of hidden units from 100 to 50, we can further trade accuracy for base calling speed. The smaller RNNs can be used in applications, where fast running times are crucial.

**Table 3:** Accuracy and running time on R9 data. The results of base calling were aligned to the reference using BWA-MEM [28]. The first column reports the percentage of reads that aligned to the reference on at least 90% of their length. The accuracy was computed as the number of matches in the alignment divided by the length of the alignment. The speed is measured in events per second.

	Aligned reads	Accuracy	Speed
Nanonet	83.2%	83.2%	2057 ev/s
DeepNano (100 hidden units)	81.1%	81.0%	4716 ev/s
DeepNano (50 hidden units)	79.3%	78.5%	7142 ev/s

**Base calling speed.** In the case of R7.3 data sets, it is hard to compare the speed of the Metrichor base caller with our base caller, since Metrichor is a cloud-based service and we do not know the exact configuration of hardware used. From the logs, we are able to ascertain that Metrichor spends approximately 0.01 seconds per event during 1D base calling. DeepNano spends 0.0003 seconds per event on our server, using one CPU thread. During 2D base call, Metrichor spends 0.02 seconds per event (either template or complement), while our base caller spends 0.0008 seconds per event. To put these numbers into perspective, base calling a read with 4,962 template and 4,344 complement events takes Metrichor 46s for template, 34s for complement, and 190s for 2D data. DeepNano can process the same read in 1.5s for template, 1.3s for complement, and 11.3 seconds for 2D data. We believe that unless Metrichor base calling is done on a highly overloaded server, our base caller has a much superior speed. Compared to Nanocall, we observed that DeepNano is 5 to 20 times faster, depending on Nanocall settings.

Although DeepNano is relatively fast in base calling, it requires extensive computation during training. The 1D networks were trained for three weeks on one CPU (with a small layer size, there was little benefit from parallelism). The 2D network was trained for three weeks on a GPU, followed by two weeks of training on a 24-CPU server, as L-BFGS performed better using multiple CPUs. Note however that once we train the model for a particular version of MinION chemistry, we can use the same parameters to base call all data sets produced by the same chemistry, as our experiments indicate that the same parameters work well for different genomes.

## Discussion and conclusions

In this paper, we have presented a new tool for base calling MinION sequencing data. Our tool provides a more accurate and computationally efficent alternative to the HMM-based methods used in the Metrichor base caller by the device manufacturer on R7.3 data. Our approach extends to R9 data, where we achieve accuracy similar to Nanonet RNN-based tool provided by Oxford Nanopore. It is also possible to trade speed for accuracy by changing number of hidden units in our RNN, according to the needs of the application.

While the architecture of DeepNano and Nanonet share some similarities, the two methods are distinct. DeepNano uses gated recurrent units, while Nanonet uses long short-term memory. Moreover, Nanonet predicts a posterior distribution over *k*-mer labels for each event, and these predictions are then combined into a single base call by additional pass through the sequence. In contrast, DeepNano’s output layer predicts for each event a bigram over a standard DNA alphabet extended with a ‘-’ symbol, which allows each event to represent zero, one, or two bases, and the result of the output layer is directly used as a basecall. We conjecture that significantly smaller output layer of DeepNano and the absence of the additional pass to assemble the predictions into the final basecall may give DeepNano an advantage in requiring smaller amount of data to train the network and being more robust with respect to calling homopolymers. However, we have not systematically evaluated these issues.

To further improve the accuracy of our tool, we could explore several well-established approaches for improving performance of neural networks. Perhaps the most obvious option is to increase the network size. However, that would require more training data to prevent overfitting, and both training and base calling would get slower.

Another typical technique for boosting the accuracy of neural networks is using an ensemble of several networks [10]. Typically, this is done by training several neural networks with different initialization and order of training samples, and then averaging their outputs. Again, this technique leads to slower base calling.

The last technique, called dark knowledge [29], trains a smaller neural network using training data generated from output probabilities of a larger network. The training target of the smaller network is to match output probability distributions of the larger network. This leads to an improved accuracy for the smaller network compared to training it directly on the training data. This approach would allow fast base calling with a small network, but the training step would be time-consuming.

Perhaps the accuracy of base calling can be futher improved by a different event segmentation or by using more features from the raw signal besides signal mean and standard deviation. We tried several options (signal kurtosis, difference between the first and second halves of the event, etc.), but the results were mixed.

At present, the DeepNano repository includes all the tools and parameters necessary for working and training on R7.3, R9, and R9.4 data. This includes event data detection functionality, replicating methods used in Nanonet. DeepNano can be used either independently of Oxford Nanopore tools, or it can use third party scaling parameters or event detection results (e.g. those generated through Nanonet or Albacore base callers).
